# Single-cell RNA sequencing reveals spatiotemporal heterogeneity and malignant progression in pancreatic neuroendocrine tumor

**DOI:** 10.7150/ijbs.61717

**Published:** 2021-08-28

**Authors:** Yu Zhou, Siyang Liu, Chao Liu, Jiabin Yang, Qing Lin, Shangyou Zheng, Changhao Chen, Quanbo Zhou, Rufu Chen

**Affiliations:** 1 Department of Pancreatic Surgery, Department of General Surgery, Guangdong Provincial People's Hospital, Guangdong Academy of Medical Sciences, Guangzhou, Guangdong, China; 2 Guangdong Lung Cancer Institute, Guangdong Provincial People's Hospital, and Guangdong Academy of Medical Sciences, Guangzhou, Guangdong, China; 3 Department of Pathology, Guangdong Provincial People's Hospital, Guangdong Academy of Medical Sciences, Guangzhou, Guangdong, China; 4 School of Medicine, South China University of Technology, Guangzhou, Guangdong, China; 5 Guangdong Provincial Key Laboratory of Malignant Tumor Epigenetics and Gene Regulation, State Key Laboratory of Oncology in South China, Sun Yat-sen Memorial Hospital, Guangzhou, Guangdong, China; 6 Department of Urology, Sun Yat-sen Memorial Hospital, Guangzhou, Guangdong, China; 7 Department of Pancreatobiliary Surgery, Sun Yat-sen Memorial Hospital, Sun Yat-sen University, Guangzhou, Guangdong, China

**Keywords:** pancreatic cancer, single-cell RNA sequencing, heterogeneity, metastatic, prognostic factors

## Abstract

**Aims:** Using Single-cell RNA sequencing (scRNA-seq), we explored the spatiotemporal heterogeneity of pancreatic neuroendocrine tumors (pNETs) and the underlying mechanism for malignant progression.

**Methods:** scRNA-seq was conducted on three tumor tissues (two primary tissues from different sites, one liver metastatic lesion), one normal liver tissue, and peripheral blood mononuclear cells from one patient with a metastatic G2 pNET, followed by bioinformatics analysis and validation in a pNETs cohort.

**Results:** The transcriptome data of 24.544 cells were obtained. We identified subpopulations of functional heterogeneity within malignant cells, immune cells, and fibroblasts. There were intra- and inter-heterogeneities of cell subpopulations for malignant cells, macrophages, T cells, and fibroblasts among all tumor sites. Cell trajectory analysis revealed several hallmarks of carcinogenesis, including the hypoxia pathway, metabolism reprogramming, and aggressive proliferation, which were activated at different stages of tumor progression. Evolutionary analysis based on mitochondrial mutations defined two dominant clones with metastatic capacity. Finally, we developed a gene signature (PCSK1 and SMOC1) defining the metastatic potential of the tumor and its prognostic value was validated in a cohort of thirty G1/G2 patients underwent surgical resection.

**Conclusions:** Our scRNA-seq analysis revealed intra- and intertumor heterogeneities in cell populations, transcriptional states, and intercellular communications among primary and metastatic lesions of pNETs. The single-cell level characterization of the spatiotemporal dynamics of malignant cell progression provided new insights into the search for potential novel prognostic biomarkers of pNETs.

## Introduction

Pancreatic neuroendocrine tumors (pNETs) are a group of heterogeneous tumors accounting for approximately 12% of primary NETs in the digestive tract and 1-2% of all pancreatic tumors.^1^ Although functional pNETs make up a dozen distinct subtypes (insulinoma, gastrinoma, glucagonoma, etc.), the non-functional pNETs have a wide spectrum of behavior ranging from indolent to highly malignant. The main prognostic factor for pNETs is the World Health Organization histological grade, which classifies pNETs as a G1, G2, or G3 tumor, or a G3 carcinoma based on the Ki-67 index, mitotic counts, and differentiation. Due to limited knowledge on the molecular mechanisms of pNET progression, treatment decisions are currently based on only the grade and stage of disease, and there are many unanswered controversies regarding optimal treatment modalities, particularly for G2 cases. Therefore, better understanding of the molecular mechanisms underlying pNET progression will inform clinical decision-making.

Genomic and transcriptomic studies have described landscapes of genetic mutations and aberrant signaling pathways in pNETs,^2^-^4^ which have significantly improved our understanding of the molecular features of pNETs. However, mechanisms underlying the development and progression pNETs is still not well understood compared to that of pancreatic ductal adenocarcinoma—therefore efficacious therapeutic approaches for patients remain elusive. Several challenges may underlie this lack of knowledge. Although pNETs are essential malignances, they often exhibit indolent biological behavior characterized by prolonged survival, especially in patients with low-grade lesions.^5^ The low incidence rate and relative indolence are challenging to both clinical study and basic translational research. In addition, pNETs have high intra- and intertumor heterogeneity, not only across patients, but also in different lesions of a single patient and have a complex tumor microenvironment (TME).^6^ Although studies based on bulk-sequencing have facilitated understanding of the molecular nature of pNETs, it is challenging to explore a heterogenous disease based on bulk mRNA sequencing.

Recently, single-cell RNA sequencing (scRNA-seq) has provided a powerful tool to characterize heterogenous cell types and has been applied to multiple cancer types.^7^,^8^ scRNA-seq of PDAC specimens revealed evolutionary mechanisms during malignant transformation of ductal epithelium and highlighted intratumoral heterogeneity in PDAC,^9^,^10^ but an scRNA-seq study in pNETs is still lacking. Thus, in this study, we employed scRNA-seq to delineate the transcriptional features of cells from both the primary lesion and the liver metastasis in a patient with a metastatic G2 pNET. We identified cell-specific transcriptional features, which enabled us to study the spatiotemporal distribution and dynamic process of each cell population within the pNET, and thereby enhanced our understanding of the mechanisms of pNET progression, which may improve clinical treatment strategies.

## Materials and Methods

### Human sample collection

A female patient with a sporadic G2 pNET was enrolled in this study. This patient had a clear history, where the pNET lesion originated at the pancreatic head, expanded to the pancreatic body, and metastasized to the liver. The patient received a pancreaticoduodenectomy and partial hepatectomy without any anticancer therapeutic prior to operation. A tumor tissue at the site of pancreatic head (T1), a tumor tissue at the distal site of pancreatic body (T2), and a pair of adjacent liver tissues (metastatic [H5] and normal [NH]) were obtained immediately after surgical removal of the specimens under the supervision of a pathologist. Fifteen ml of peripheral venous blood was collected on the same day of, but prior to, the operation. This study was approved by the Ethics Committee of Guangdong Provincial People's Hospital (IRB number KY2020-359-01).

A Complete description of methods was attached in Supplementary Methods.

## Results

### Sample acquisition and cell lineage determination

**Figure 1:**
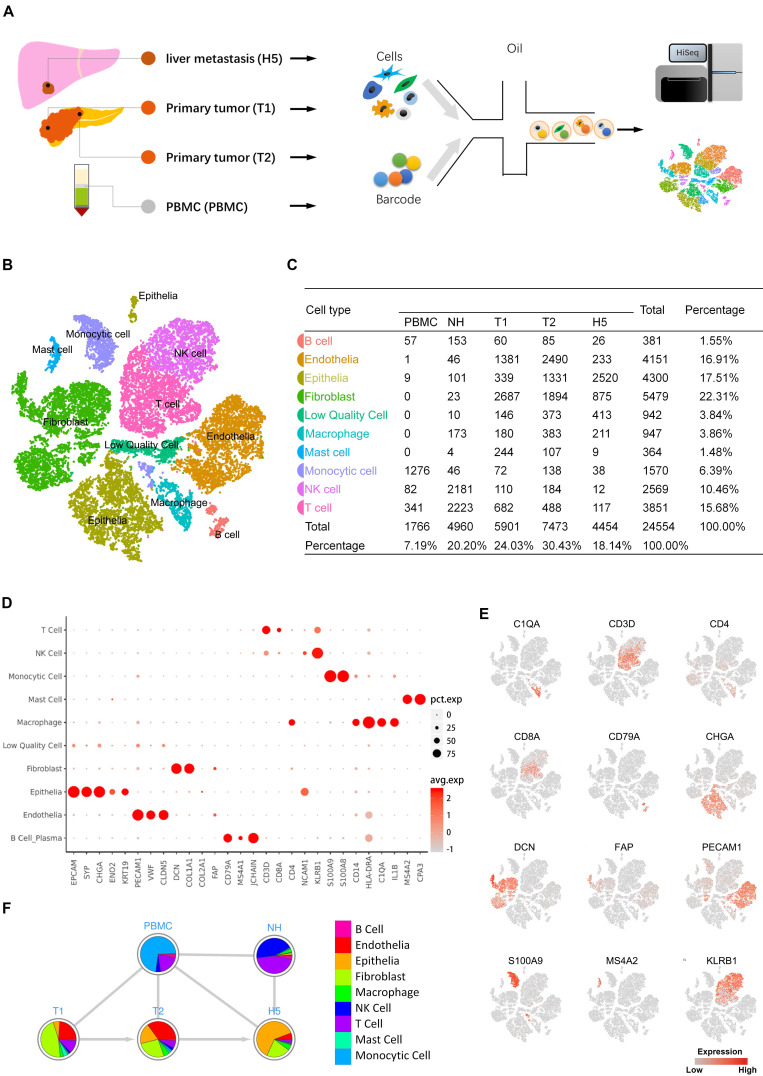
A. Scheme of the overall study design. Single-cell RNA sequencing was applied to cells derived from a total of four samples (PBMC 1, primary tumor 2, metastasis 1, and normal liver tissue 1), and the output data were used for bioinformatic analyses and verification in tissue. B. The tSNE projection of 24,544 cells from 4 samples, showing the formation of 10 main clusters. Each dot corresponds to one single cell, colored according to cell cluster. C. Cell number and percentage of assigned cell types are summarized. D. Dot plot demonstrates the normalized mean expression of specific markers in each cell cluster. E. Expression levels of the indicated marker genes are projected onto the tSNE map. F. Pie charts represent the proportions of cell types in each sample source. pct.exp, percentage of cells with expression; avg.exp, average expression level.

To explore the transcriptomic states of individual cells in both primary and metastatic lesions of pNETs, we isolated cells from the primary tumor, metastatic liver tumor, normal liver, and peripheral blood from a treatment-naïve patient with metastatic pNETs and performed scRNA-seq (Figure 1A). Data originating from 24,544 cells were retained for downstream bioinformatics analysis (Supplementary Table S1). Cell lineage determination was done using a PCA. We employed tSNE and UMAP to reduce the dimensionality of this data and allow the visualization of cell-type clusters defined by their transcriptional profiles. As a result, a total of nine main cell clusters were identified, including epithelia, endothelia, fibroblast, B cell, monocytic cell, macrophage, mast cell, NK cell, and T cell (Figure 1B, 1C). We then generated cluster-specific marker genes by performing differential gene expression analysis, which identified mutually exclusive gene sets including established markers of particular cell types (Figure 1D, E). Pie charts indicated the percentage of each cellular components in each sample (Figure 1F). Notably, the cell composition from different sources showed variability, even in tumor tissues.

### Characterizing the heterogeneity in malignant cells

**Figure 2:**
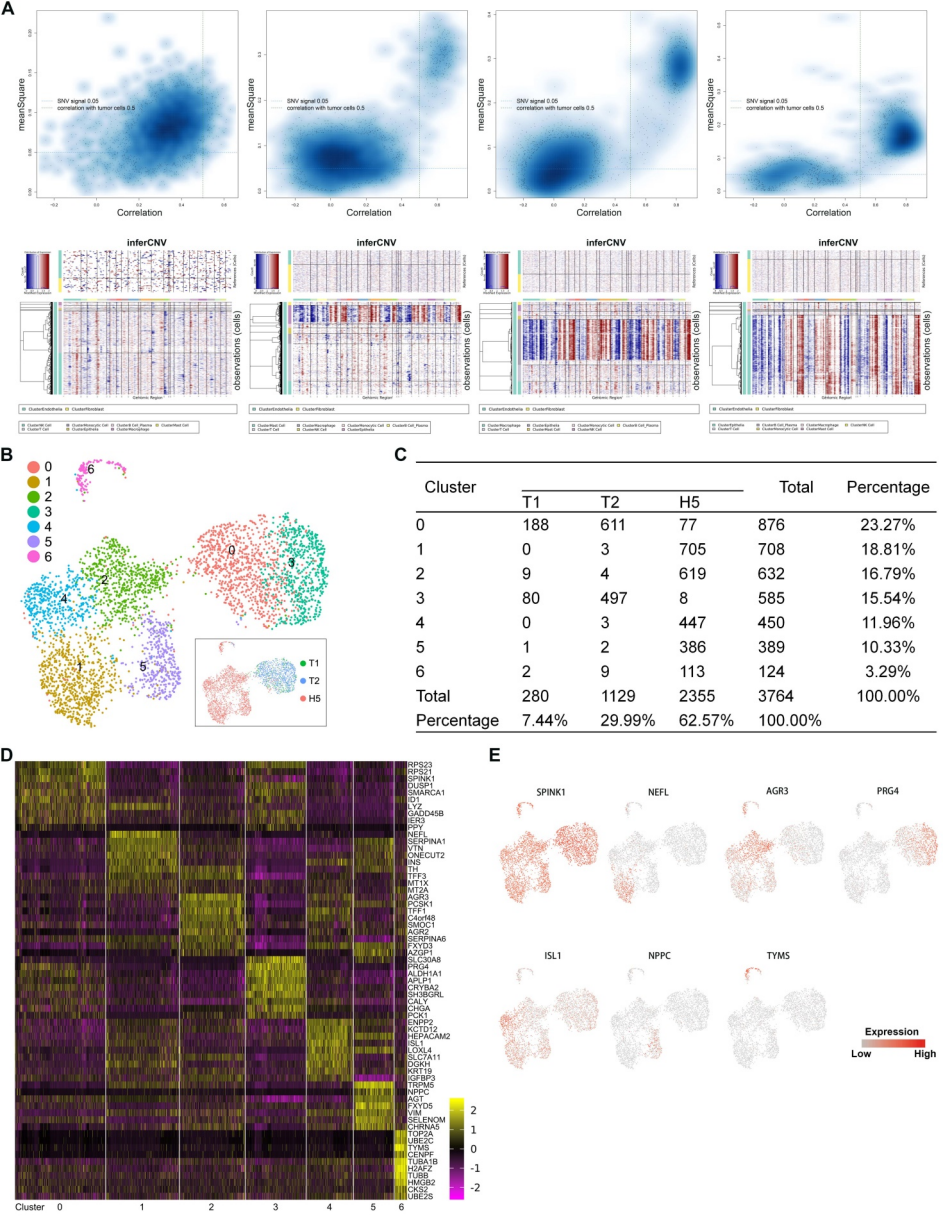
A. Quadrant plots showing the selection of malignant cells were displayed in the upper panels. The y-axis represents the CNV signal and the x-axis represents the CNV correlation. Each dot represents a single cell. The cell population in the upper right quadrant was defined as the putative malignant cell population (CNV signal above 0.05 and CNV correlation above 0.5). Heatmaps of large-scale CNV events in individual cells for each sample are shown in the lower panels, in which the y-axis represents each cell, and the x-axis is the genomic position of CNV events. Red: amplifications; blue: deletions. B. UMAP representation of malignant cells following graph-based clustering colored in seven subclusters. The inset in the black box is a UMAP picture colored by the source of cells. C. Cell numbers of each subcluster are summarized. D. Heatmap showing expression levels of specific markers in each cell type. E. Expression levels of the indicated marker genes projected onto UMAP plots.

To investigate the heterogeneity in malignant cells, we first calculated large-scale chromosomal copy number variation (CNV) in each cell in order to define malignant cells (Figure 2A) (Supplementary Table S2). A total of 3,746 malignant cells were retained. To reveal the functional subtypes, the malignant cells were subclustered by PCA and visualized by UMAP projection. A total of 7 stable clusters emerged (Figure 2B, 2C), named from subtype 0 to subtype 6. Sub-cluster-specific markers for each subtype were shown in the heatmap (Figure 2D). We also identified multiple markers to distinguish different malignant subtypes (Figure 2E). Significant heterogeneity in the composition of the 7 subtypes was found between the primary and metastatic tumor. The primary tumors comprised predominantly two subtypes, 0 and 3. Subtype 3 seemed to be a malignant population that was dominant in primary sites. Compared with primary lesions, a higher diversity was found in the metastatic tumor, which contained all subtypes except subtype 3.

**Figure 3:**
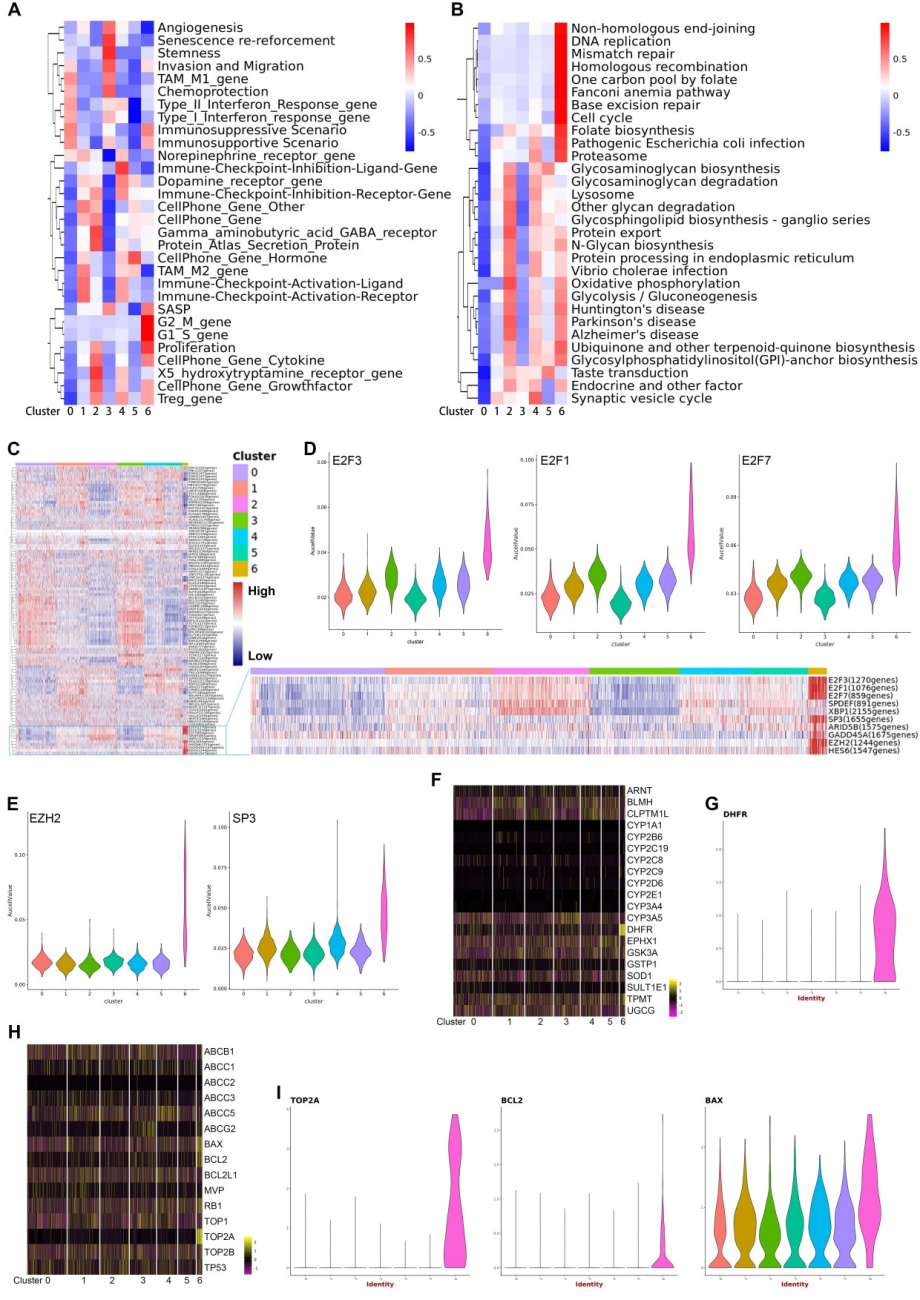
A, B. QuSAGE Gene Enrichment Analysis revealed enriched biological processes (A) and KEGG pathways (B) for specific genes of each malignant subcluster. C. Heatmap of SCENIC analysis, showing the relative activity of transcription factors in each malignant subcluster at single-cell level. The blue box highlights the prominent transcription factors with high activity specific to subcluster 6. D. Violin plots displaying the expression of E2F3, E2F1, and E2F7 across the seven malignant subclusters. E. Violin plots displaying the expression of EZH2 and SP3 across the seven malignant subclusters. F. Heatmap showing the expression levels of drug-metabolism genes in each malignant subcluster. G. A violin plot showing the expression of DHFR in each malignant subcluster. H. Heatmap showing the expression levels of drug-resistance genes in each malignant subclusters. I. Violin plots showing the expression of TOP2A, BCL2, and BAX in each malignant subcluster.

The finding that each subtype expresses a specific gene set that can be used for distinguishing these subgroups indicated there were different biological characteristics in different subtypes of malignant cells. To characterize the relative activation of primary cellular functions, we performed QuSAGE analysis to determine the enrichment level of well-known gene sets for each subtype and found that each subtype had unique functions (Figure 3A, 3B) (Supplementary Table S3). Notably, the significantly enriched cellular functions in subtype 6 were related to biosynthesis, mitosis, cell cycle, and cell proliferation, which was further supported by the enriched expression of *CDK* and *CDKN1* family genes. In addition, enrichment of gene sets related with angiogenesis and stemness were observed exclusively in subtype 3. Subtype 4 was enriched for immune-checkpoint inhibition. In contrast, genes in subtype 1 were enriched for immune-checkpoint activation, suggesting a possible association with immune response in these two subtypes. Additionally, enriched gene sets specific for subtypes 2 and 5 were mainly related to GABA signaling and cell-cell communications via hormones.

To reveal the driving mechanisms for the functional divergence in each subtype, we performed SCENIC (Single cell regulatory network inference and clustering) analysis to delineate transcriptional dynamics of the malignant cells and to reveal potential transcription factors underlying the regulation across subtypes at single-cell level (Supplementary Table S4). Hierarchical clustering of the activity of transcriptional factors by SCENIC across the 7 subtypes is shown in a heatmap (Figure 3C). In particular, subtype 6 showed significantly increased expression of three E2F family members (*E2F1*, *E2F2*, and *E2F3*), *EZH2*, and *SP3*, which all were well-known oncogenes participating in cell proliferation, differentiation, and invasion (Figure 3D, 3E).^11^-^14^ On the other side, although curative resection is the primary treatment choice for pNETs, antineoplastic agents are essential for the treatment of most pNETs. Therefore, we analyzed the expression of genes involved in drug resistance and drug metabolism (Figure 3F-3I). As shown in the heatmaps, the expression of *DHFR*, *BAX*, and *TOP2A* in subtype 6 were significantly different from the other subtypes. Violin plots show the exclusive expression of *DHFR*, *BCL2,* and *TOP2A* in subtype 6, as well as significantly increased *BAX* expression. *DHFR*, *TOP2A*, and *BCL2* were unfavorable biomarkers that predict chemoresistance to temozolomide,^15^-^17^ an alkylating agent, used as a chemotherapy agent in patients with pNETs.^18^,^19^ Additionally, inhibition of BCL2 seemed a way to enhance the response to everolimus,^20^ a mTOR inhibitor showed effect in pNETs. Interestingly, Bax expression was correlated with sensitivity to capecitabine,^21^ an agent used as a monotherapy or usually combined with other chemotherapy agents.^22^ These findings showed the acquisition of heterogeneity and diversity in drug-resistance/sensitivity during the evolution of pNETs, and subtype 6 was a malignant subpopulation with high proliferation activity and drug-resistant ability.

Compared with other subtypes, the subtype 3 seemed a malignant population that was dominant in primary sites. In the above QuSAGE functional annotation analysis, we have observed the gene sets related with angiogenesis and stemness, two important hallmarks in carcinogenesis, were enriched in subtype 3. To better understand the heterogeneity of pNETs, we further investigated the characteristics of subtype 3. Additional QuSAGE analysis was performed by using a customized gene set including immune-, cytokine- and neurobiology-related terms.^23^ As shown in Supplementary Figure S1A, the gene signature of “neuroendocrine” was specifically enriched in subtype 3. Neuroendocrine tumors (NETs) are malignant growths originating from neuroendocrine cells, therefore, the enrichment of neuroendocrine gene signature suggested that the subtype 3 displayed more genes characteristic of the cell of origin in comparison with other subtypes. The trajectory analysis using all malignant cells further revealed that the subtype 3 was a subpopulation at the initial phase of malignant evolution (Supplementary Figure S1B).

Gene set enrichment analysis (GSEA) also showed significant enrichment of genes related to neuroendocrine cell differentiation in subtype 3 (Supplementary Figure S1C), but the difference in the enrichment of signature genes related with neuroendocrine neoplasm was not significant between subtype 3 and other malignant subtypes, which further supported that, among all malignant cells, the subtype 3 was an initial subpopulation with most features of the neuroendocrine cells from which they are derived. In terms of transcriptional regulation, the SCENIC analysis on all malignant subtypes showed specific enhanced activity of TCF4 (transcription factor 4) in the subtype 3 (Supplementary Figure S1D). It is well known that TCF4 is a gene which was found to be abundantly expressed during neural development,^24^ and TCF4 was also involved in neuroendocrine differentiation of tumor cells,^25^ which was consistent with the above findings about the prominent feature of neuroendocrine in subtype 3. Additionally, TCF4 has been found to be associated with tumorigenesis in a variety of tumors and was a potential molecular target against kinds of cancer,^26^-^29^ suggesting that TCF4 may be an intervention target for the subgroup 3.

The other two functional characters of angiogenesis and stemness revealed by functional annotation analysis in subtype 3 were subsequently investigated. As mentioned in the supplementary method, a systematic search of electronic databases for the cancer stem cell markers of neuroendocrine tumors was performed, a total of fifteen markers were found to have been reported previously (Supplementary Table S5), in which we found four markers were reported in more than one study, including ALDH1, CD133, CD44, and SOX9. Consequently, expression levels of the classical neural stem cell marker Nestin (NES),^30^ as well as the above four markers were evaluated across all malignant subtypes. As shown in Supplementary Figure S1E, NES was expressed in both two subtypes of primary tumor where subtype 3 predominate, which could be explained, at least in part, that subtype 0 and subtype 3 were two subpopulations located at the early phage of tumor evolution and they may share some gene features with the neural stem cell which they may be derived from. In addition, the expression of ALDH1, CD133, CD44 and SOX9 was heterogeneous among the seven subtypes. Co-expression of ALDH1 and CD133 was found in the subtype 3, but the expression of CD44 and SOX9 was low in subtype 3. None of the subtypes showed uniformly high expression of all cancer stem cell markers, revealing probable heterogeneity in the expression of cancer stem cell markers at single-cell level. Moreover, GSEA showed significant enrichment of angiogenesis-related gene sets in cluster 3 in comparison with other malignant subtypes (Supplementary Figure S1F); additionally, as shown in the dot plot of Supplementary Figure S1G, subtype 3 showed increased expression of angiogenic TGFB1, VEGFA, and VEGFC than other subtypes. Therefore, the cells of subtype 3 may play a significant role in angiogenesis through releasing angiogenic factors.

### Intra- and intertumoral heterogeneities of immune cells and fibroblasts

We performed the subclustering of myeloid cells to help understand the relationships between subpopulations and their functions, and we detected 11 subsets Supplementary Figure S2A, B). Monocytes, macrophages, and dendritic cells were defined by well-known markers (Supplementary Figure S2C), and we found 5 subsets of macrophages (Supplementary Figure S2A-C). The macrophage-1 subset was located predominantly in primary tumors, while macrophage-2 and macrophage-5 subsets existed exclusively in liver metastasis. Different expression of markers for macrophage subtypes, including *M1*, *M2a*, *M2b*, *M2c*, and *TAM*,^31^ are displayed in a dot plot (Supplementary Figure S2D). The expression of M1/M2 signatures in the five macrophage subsets we observed did not fall in line with either the canonical M1 or M2 classifications. Clusters also showed differential enrichment of hallmark gene-set activity, confirming their distinct transcriptional programs (Supplementary Figure S2E, S2F), in which we found the macrophage-1 showed abnormal activation of most M2 macrophage-related pathways. Specifically, macrophage-1 was the subset closest to M2 phenotype, but this subset still expressed some signature genes of the M1 phenotype. In contrast, the liver metastasis-specific macrophage-2 showed more M1 characteristics. Since macrophages mainly affect tumor cells by paracrine chemokines and cytokines, we further analyzed the expression of cytokines and chemokines across both macrophage-1 and macrophage-2 subsets (Supplementary Figure S2G). We found that *CCL2*, the dominant chemokine for the migration of tumor-promoting MDSC and regulatory T cells,^32^ was expressed in macrophage-1 and macrophage-2 subsets. Additionally, *CCL3* and *CCL4* were also expressed mainly in macrophage-1 and macrophage-2 subsets at similar levels. Both *CCL3* and *CCL4* are double-edged chemokines that exert antitumor and protumor behaviors, which depended on the microenvironment.^33^,^34^ However, *CCL13*, a chemokine that is a chemoattractant for eosinophils, basophils, monocytes, macrophages, immature dendritic cells, and T cells,^35^ was exclusively expressed in the macrophage-1 subset (Supplementary Figure S2G, S2H). In addition, *SPP1*, of which the product is related to a fibroblastic microenvironment and supports monocyte/macrophage proliferation,^36^-^38^ was specifically expressed in macrophage-2 and macrophage-5 subsets but was not found in primary tumor subsets (Supplementary Figure S2G, S2H).

To delineate the diversity of CD8^+^ T cells within the TME, we selected cells with known markers *CD3* and *CD8A* and identified seven clusters (Supplementary Figure S3A, S3B). Clusters 4 and 5 were the major subsets in primary tumors, and cluster 4 was almost exclusively found in primary tumors. Notably, we found only a few CD8^+^ T cells in liver metastasis. To further address the features of subclusters, the expression of markers of T cell subtypes were explored. We examined each cluster for the expression of effector memory markers (*FCGR3A*, *CX3CR1*, *FGFBP2*, *GNLY*), MAIT (mucosa-associated lymphoid tissue) markers (*CD127*, *CD161*, *SLC4A10*), cytotoxic genes (*GZMA*, *GZMB*, *GZMK*), and exhausted marker *DUSP4* (Supplementary Figure S3C).^39^,^40^ Cluster 4 appeared to be the “exhausted T cells”, which was also supported by the result of functional enrichment analysis that the activity of immunoreactive pathways was lowest in cluster 4 (Supplementary Figure S3D). However, the absence of classical inhibitory receptors such as *PDCD1* and *CTLA4* suggested there exists another immunosuppressive mechanism (Supplementary Figure S3E). Using RNA velocity, a method inferring cell dynamics,^41^ we observed a clear directional flow to cluster 4 (Supplementary Figure S3F), further suggesting these were the exhausted T cells. Low expression of *PRF1* as well as most of the cytotoxic genes were found in cluster 4 (Supplementary Figure S3G). Therefore, cluster 4 seemed closer to the exhaustion state. A previous study reported that expression of PD-1 or PD-L1 in the small intestine or pNET was rare and low,^42^ therefore it is not surprising that we did not find typical exhausted T cell (Tex) in the present analysis.

Cancer-associated fibroblasts (CAF) are major players in the progression of multiple solid tumors.^43^ Therefore, subclustering of fibroblasts was performed and we identified a total of 14 clusters (Supplementary Figure S4A, S4B). Overall, the TME of primary tumors contained more heterogeneous clusters of fibroblasts than TME of liver metastasis. Previous studies suggested there were three kinds of CAF in TME of pancreatic cancer: the inflammatory CAFs (iCAF), antigen-presenting CAFs (apCAF), and myofibroblastic CAFs (myCAF).^44^,^45^ We evaluated the expression of markers for the three kinds of CAFs at the single-cell level across each cluster (Supplementary Figure S4C). Based on the differences in the expression of these markers, we found that eight clusters were associated with enrichment of iCAF markers, and four clusters were associated with enrichment of myCAF markers (Supplementary Figure S4D, S4E). Among them, the cluster 5 was classical iCAF, and the cluster 8 was classical myCAF, and both were located exclusively in primary sites. Moreover, cluster 6 was defined as apCAF due to its high expression of apCAF markers, such as *CD74*, *CD200*, and human leucocyte antigens (MHC class II chains [*HLA-DRA*, *HLA-DRB1*, and *HLA-DPA1*]; Supplementary Figure S4F). We identified multiple immune-related pathways enriched in the subset 6, including antigen-presentation (Supplementary Figure S4G). We found for the first time a presence of the apCAF signature in pNETs. The presence of MHC class II molecules suggests that this fibroblast subset can interact with CD4^+^ T cells, but the high expression of CD200, a newly identified immune checkpoint protein, suggests that this fibroblast subset plays a different role compared to professional antigen-presenting cells.

Cellular senescence plays a critical role in tumorigenesis.^46^ As shown in the bubble plot displaying the expression of senescence-associated secretory phenotype (SASP) genes (Supplementary Figure S4H), clusters 5 and 7 were senescent fibroblasts secreting more protumorigenic SASP factors, such as *IL6*, *CXCL12*, *MMP*s, and *VEGF*s. However, we noticed that *CCL2*—the dominant chemokine gene for the migration of MDSC—was higher in clusters 0 and 3, while another proinflammatory cytokine *CCL5* was uniquely expressed in cluster 13. Clusters 4 and 10 were specifically located in the liver metastasis. Unlike in other fibroblast clusters of the primary tumor, low levels of most traditional protumorigenic factors were observed in subsets 4 and 10. However, high expression of oncogenic factors including *IGFBP2*, *PGF*, and *TGFB2* was found specifically in subset 4, suggesting a different functional role of fibroblasts in liver metastasis compared to the primary tumor. Collectively, these findings suggested the development of functional diversity during the multidirectional differentiation of fibroblasts.

### Dynamic landscape of single-cell transcriptome

**Figure 4:**
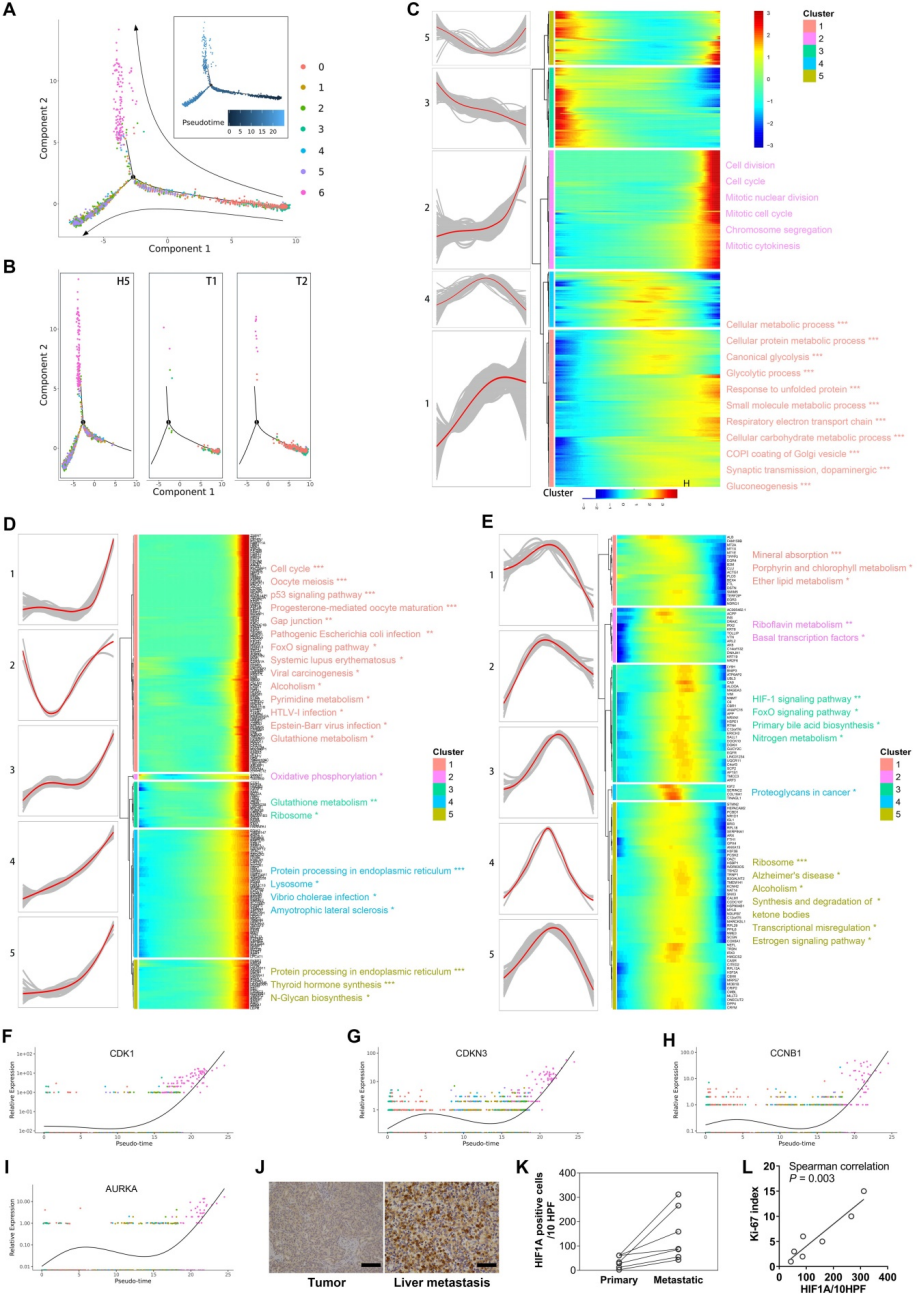
A. Prediction of malignant developmental trajectory with subcluster and pseudo-time information mapped on, and each point corresponds to a single cell. B. Malignant developmental trajectories of single malignant cells for each tumor sample. Subcluster information is also shown. C. Heatmap showing expression of differentially expressed genes (rows) ordered by pseudo-time (columns). Color key from blue to red indicates pseudo-time from initiation to end. Five patterns of gene expression along the pseudo-time are displayed in the left panel. The top significantly enriched biological processes were summarized at the right of the corresponding gene cluster. *, P < 0.05; **, P < 0.01; ***, P < 0.001. D, E. The genes of patterns 2 and 4 mentioned in (C) were further clustered hierarchically. The more detailed subpatterns of pattern 2 (D) and 4 (E) were obtained. Similar to (C), the subpatterns, genes ordered by pseudo-time, and the top significantly enriched biological processes are shown. F-I. The relative expression of cell-cycle related genes in each malignant subcluster along the pseudo-time are plotted. J. Representative IHC images of HIF1A protein expression in primary site and liver metastatic site are shown. K. The dot-line plot shows the change in the number of HIF1A positive cells per high-power field (HPF) between paired primary and liver metastatic lesions. L. Correlation between the number of HIF1A-positive cells per HPF and Ki-67 index in seven liver metastatic lesions was estimated by Spearman's correlation analysis.

It is not yet clear how tumor cells of pNETs evolve from the initiation, progression, to metastasis. To investigate the evolutionary process during pNETs progression, we performed the trajectory analysis using malignant cells. Figure 4A visualizes the evolutionary trajectories by reconstituting all malignant cells. The distribution of malignant cells along the trajectory in each sample source (Figure 4B) was shown. Consistent with the actual situation of tumor sites, cells from primary sites were located mostly at the start site, and cells from the liver metastasis rarely appeared at the start site. In particular, cells of subtype 6 were distributed at the extreme end site. Given the abnormal enrichment of pathways involved in cell cycle, cell proliferation, and differentiation in subtype 6, it is reasonable to speculate that the subtype 6 is a population of highly malignant cells produced in the late stage of tumor evolution.

We next focused on the gene expression patterns along the process of malignant cell evolution. We studied the dynamics of malignant evolution by generating a profile of gene expression changes across the pseudo-time trajectory. The changes followed five patterns (Figure 4C) (Supplementary Table S6). Genes in patterns 1 and 2 were upregulated, and functional annotation revealed that they were correlated with metabolism (e.g., Oxidative phosphorylation, Glycolysis/Gluconeogenesis, Carbon metabolism, Pyruvate metabolism, and Glutathione metabolism), biosynthesis and degradation (e.g., Biosynthesis of amino acids, Protein processing, and Lysosome), and cell proliferation (e.g., Cell cycle and Oocyte meiosis), suggesting both the level of metabolic reprogramming and the ability of cell proliferation increased with tumor progression. Further clustering the expression patterns of genes in patterns 2 (Figure 4D) and 4 (Figure 4E) revealed more detailed dynamic trends. Genes in subpatterns 1 and 2 showed a sharply increased expression level at the late stage; most of them were associated with cell cycle, cell division, and mitosis, indicating the gain of rapidly proliferative capability was a sudden late event along cell evolution (Figure 4D). The single-cell level expression of *CDK1*, *CDNK3*, *CCNB1*, and *AURKA*, genes involved in the cell cycle and cell proliferation, along the pseudo-time trajectory is displayed in Figure 4F-4I. We found a sharp increase of these genes at the end-stage of the trajectory, especially in cluster 6 of malignant cells.

Most studies tried to explore key genes involved in tumor progression through analyzing differentially expressed genes, largely based on the hypothesis that tumors exhibit different malignant biological behavior through differential gene expression.^47^ Interestingly, we witnessed that some genes stayed at similar expression levels at early and late stages, but displayed significantly altered expression at the intermediate stage. Functional analysis demonstrated that genes of pattern 4, those that displayed upregulation in cells at the intermediate stage, were associated with blood component-related pathways, such as platelet degranulation, cellular response to erythropoietin, response to triglyceride, platelet activation, and blood coagulation. Further detailed analysis showed that the genes in the subpattern 4 of pattern 4 were associated with proteoglycans, which is a component of cell membrane or extracellular matrix that participates in cell migration and invasion. These results suggest that cells in the intermediate stage are of peri-metastasis status. Notably, the genes in subpattern 3 of pattern 4 represented the most typical pattern of high-expression at the intermediate-stage. These genes were correlated with the HIF-signaling pathway, indicating the HIF pathway played a more important role in the intermediate stage that in early or late stages. We performed immunostaining for HIF-1α in paired primary tumor (n=5) and liver metastasis (n=7), and found increased expression of HIF-1α at the liver metastasis (Figure 4J, 4K). In addition, expression of HIF-1α was associated with Ki-67 index (Spearman's correlation P = 0.003, Figure 4L), suggesting HIF-1α was involved in the proliferation of tumor cells.

### Evolutionary analysis for malignant cells

**Figure 5:**
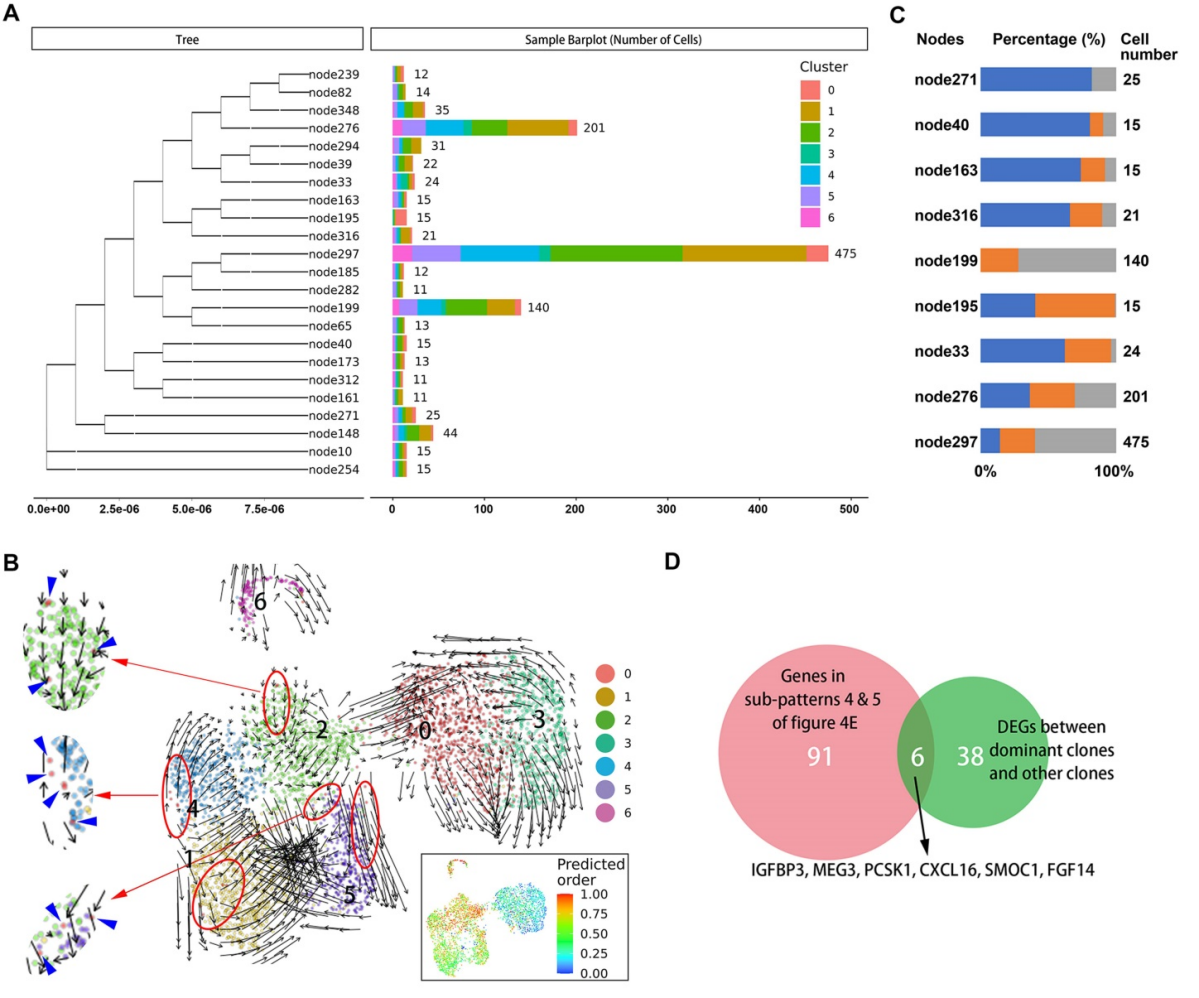
A. Branches of the mitochondrial phylogenetic tree of malignant cells. Cell numbers in each branch, as well as the clusters information are shown. B. RNA velocities are visualized on the UMAP projection with clusters information of malignant cells. The inset plot (in the black box): CytoTRACE results were visualized on the UMAP projection, showing the predicted developmental order of cells; color key from blue to red indicates pseudo-time from initiation to end. Blue arrows highlighted the distributed cells of cluster 0. C. Components of cell source in main branches were estimated and summarized in the bar chart. Total number of malignant cells in each site [primary tumor 1 (T1), primary tumor 2 (T2), liver metastasis (H5)] was used as reference for normalizing the cell proportion of corresponding sites in each branch. Blue bar, primary tumor 1 (T1); orange bar, primary tumor 2 (T2); gray bar, liver metastasis (H5). D. Venn plot showing the intersection of genes steady increase during tumor progression and genes significantly dysregulated in dominant clones.

It is not known which tumors cells are ready and responsible for metastasis in the primary site. Therefore, we sought to identify characteristics of primary tumor cells with metastatic potential. First, we traced the lineage origins of metastatic cells by analyzing the mitochondrial mutations. As shown in Figure 5A, the phylogenetic tree of malignant cells constructed based on mitochondrial mutations identified a dozen lineages including both primary and metastatic cells, suggesting a single metastatic lesion contained multiple cells of origin. Each lineage also contained multiple malignant subclusters, indicating a seed cell has the differentiation potential towards different biological characteristics. We further performed RNA velocity analysis of malignant subsets and observed that the cells of cluster 0 distributed as the starting point in all metastatic-specific clusters (clusters 1, 2, 4, 5; Figure 5B), supporting the multicell origin of liver metastasis, as well as the multidirectional differentiation potential of metastatic primary tumor cells.

The origins of cells in several dominant lineages were analyzed, and the proportions of cell origins in each lineage were normalized according to the total number of malignant cells in the corresponding tissues (Figure 5C). We selected three nodes, node276, node297, and node199, in which the numbers of tumor cells were significantly higher than in other nodes, as the dominant clones with metastatic potential and proliferative advantage in the liver metastatic niche. Accordingly, the differentially expressed genes between the dominant clones and others were estimated, and their intersection with genes in subpatterns 4 and 5 (Figure 5D), which represented the genes that continuously increase during cell progress from primary site to metastasis, was obtained. Overall, we identified five differentially expressed protein-coding genes (*IGFBP3*, *PCSK1*, *CXCL16*, *SMOC1*, and *FGF14*) (Supplementary Table S7).

### Identification of markers of relapse risk

**Figure 6:**
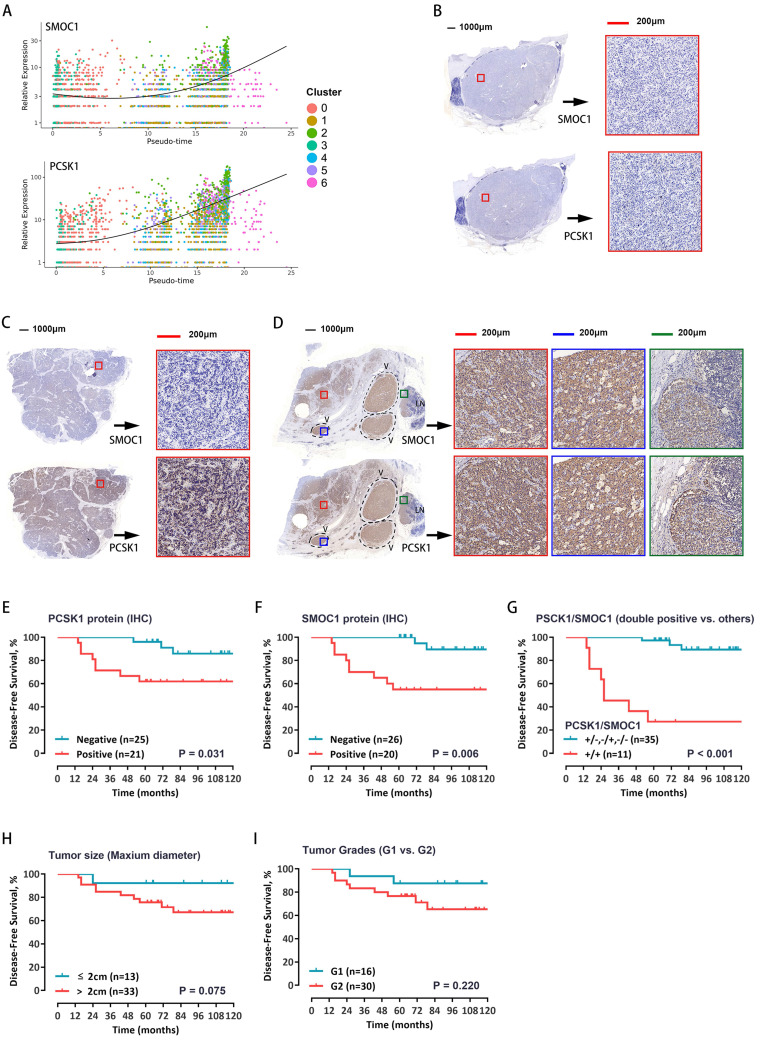
A. The relative expression of SMOC1 and PCSK1 in malignant cells along the pseudo-time are plotted. Each dot corresponds to one single cell, colored according to cluster of malignant cells. B-D. Immunohistochemical analysis for SMOC1 and PCSK1 was carried out using continuous paraffin sections. Representative results of double-negative (B), single-positive (C), and double-positive (D) are shown. E, F. Kaplan-Meier curves with log-rank tests for the disease-free survival of the 46 patients with low vs. high immunohistochemical protein expression of PCSK1 (E) and SMOC1 (F). G. The Kaplan-Meier curves with log-rank tests for the disease-free survival of the 46 patients with double positive for PCSK1 and SMOC1 vs. others. H, I. Patients were stratified by tumor size (H) or WHO grade (I), and the disease-free survival between groups was estimated by Kaplan-Meier curves with log-rank tests.

To test whether these five genes can be used as prognostic markers in patients with pNETs, the RNA expression levels were evaluated in a total of 46 pNET patients (Supplementary Table S8). Among the above five genes, survival curves showed that both *PCSK1* and *SMOC1* expression levels have much closer relationship with the recurrence, the expression of *PCSK1* and *SMOC1* was significantly correlated with prognosis (Supplementary Figure S5A-E). The single-cell expression levels of *PCSK1* and *SMOC1* along cell progression are shown in Figure 6A. Further, the protein expression levels of *PCSK1* and *SMOC1* were detected by immunostaining—divided into positive or negative expression. Representative images of double-negative, single-positive, and double-positive immunohistochemical staining are shown in Figure 6B-D, respectively. Survival curves showed that both *PCSK1* and *SMOC1* protein expression levels were significantly correlated with the recurrence (Figure 6E, 6F), and double-positive *PCSK1*/*SMOC1* protein expression was the more unfavorable prognostic factor (Figure 6G) than a single marker, tumor size (Figure 6H), and tumor grade (Figure 6I). These findings suggest that double-positive expression of PCSK1 and SMOC1 may identify high-risk patients with postoperative recurrence.

## Discussion

pNETs have been increasingly diagnosed and resected during recent decades.^48^ However, the high heterogeneity of pNETs leads to various biological behaviors and clinical outcomes, which poses tremendous challenges for the clinical management of pNETs after surgical resection. Thus, it is highly desirable to explore the intra- and intertumoral heterogeneity and underlying mechanisms, to better improve the prognosis of pNETs. Compared with the highly aggressive G3 tumors, more treatment modalities are used in G1/G2 pNETs. These well-differentiated G1/G2 tumors are associated with relatively indolent physiological behavior. For this reason, only a few studies have investigated the factors associated with recurrence in this group of patients. Although there were traditional pathological indices such as tumor size, Ki-67, mitotic count, and lymphatic metastasis, the actual predictors of survival and recurrence after Pan-NEN resection are still controversial, and there is a lack of molecular markers to guide therapeutic selection. By using the scRNA-seq technique, we demonstrated that tumor progression leads to a series of dramatic cellular changes in both composition and functional state. Specifically, we analyzed the dynamic changes in gene profiles of malignant cells from primary state to metastasis, and further developed a two-gene prognostic signature.

Malignant cells identified by CNV-based analysis were divided into several subclusters with functional heterogeneity by comparing gene expression levels. Differences in the composition of malignant subclusters were observed among tumors from different origins. Our pseudo-temporal ordering analyses revealed the hypoxia-related mechanism and metabolic reprogramming bridges the process of tumor development from primary site to metastatic lesion. Notably, a subcluster (the malignant subtype 6) with highly enhanced proliferative potential was exclusively detected in the liver metastasis, suggesting it may emerge along tumor progression. Pseudo-time analysis also revealed that this cluster was a group of highly differentiated malignant cells appearing at the terminal stage of the tumor evolutionary process. Interestingly, several drug-resistance genes were specifically expressed in this cluster. Since the samples were from a treatment-naïve case, it is reasonable to infer that pNETs can acquire drug-resistance without drug selective pressures. Thus, adequate evaluation of the drug-resistance characteristics must be performed in both primary and metastatic lesions. In contrast to the subtype 6, the subtype 3 seemed a subpopulation located at the initial phase of malignant evolution, which was supported by enrichment of gene signatures related with neuroendocrine cells and the marker of neuro stem cells, both were the supposed source of neuroendocrine neoplasms. Although the subtype 3 was present almost exclusively in the primary tumors, functional enrichment analysis revealed this subtype may be the predominant angiogenesis-promoting malignant subpopulation. These present results advance our current understanding of the tumor cell heterogeneity of pNETs.

The immune microenvironment was quite different between the primary and metastatic lesions. Some subpopulations of immune cells were location-specific. We discovered that gene expression profiles for TME macrophages were heterogenous and did not fully meet the classic M1/M2 phenotypes. The M1/M2 macrophage polarization paradigm was introduced by Charlie Mills in 2000.^49^ This concept is an in vitro construction that is built on a stimulation of macrophages with a defined set of cytokines. It was challenged by emerging studies for its applicability in the TME.^50^,^51^ Single-cell evidence in some malignancies have revealed that macrophages in the TME are not confined to a binary M1/M2 designation.^52^,^53^ In macrophage biology of TME, a better understanding of the all-encompassing spectrum bridging the two extreme phenotypes was required. Like macrophages, the classical “exhausted T cells” were not defined in our analysis. Low expression of classic immune checkpoint receptors was observed, while several other inhibitory checkpoints, such as *CD44* and *SIRPA*, were elevated in some subsets of CD8^+^ T cells. Moreover, we found very few T cells in the liver metastatic lesion, suggesting the cytotoxic immune microenvironment could be different between primary and metastatic tumors. Although our findings were in accordance with a report that expression of PD-1 or PD-L1 in pNETs was rare, a limitation of our analysis is that the cells were derived from one patient. Further studies to define the comprehensive immune checkpoint profiles of both primary and metastatic lesions are warranted.

In PDAC, it is well known that the TME comprises a heterogeneous population of CAFs with different functions,^54^ but the CAF populations in pNETs remains unclear. Our present study revealed that, like other stromal cells, fibroblasts also exhibited a wide heterogeneity in terms of functional states and distributions. A previous study suggested two subtypes of CAFs in PDAC: CAFs expressing high levels of α-SMA, which we named myCAFs, and CAFs expressing low levels of α-SMA, but high levels of cytokines and chemokines, which we named iCAFs.^44^ A recent single-cell analysis of PDAC corroborated the presence of myCAFs and iCAFs and defined their unique gene signatures in vivo.^45^ According to the signatures proposed in this study, we identified two subclusters in which the expression of signature genes aligns with the classic iCAFs and myCAFs. However, most subclusters did not fully align with the signatures. Interestingly, in two subsets specific to liver metastasis, subsets 4 and 10, we observed that subset 4 had higher levels of *α-SMA* and cytokines compared to subset 10, which was opposite to the iCAF/myCAFs definition. We discovered that fibroblasts of pNETs had gene expression profiles of other markers, especially the genes encoding functional cytokines, and were different from that in the PDAC, suggesting there are differences in the subpopulations and functional roles between fibroblasts in pNETs and in PDAC. Additionally, we identified a subpopulation of fibroblasts expressing specific markers of apCAF. The presence of human leukocyte antigen on this subpopulation suggests a potential interaction with T cells;^55^ however, since this subpopulation had the highest expression of immune checkpoint inhibitors among all subpopulations of fibroblasts, it seems that this apCAF subpopulation might preferentially cause immunosuppression, but not immune activation.

Using scRNA-seq, we described the evolutionary trajectory of malignant cells at the single-cell level. Several key molecular events in the process of tumor progression from primary to metastatic lesions were identified. For example, we found the metabolic reprogramming developed at the “mid-late” stage, this also supports the concept that tumor metabolism is dynamic and adaptable to the TME. Meanwhile, the extensive proliferation capacity was suddenly acquired along tumor evolution, which happened in the liver metastasis. Previous studies support that primary and metastatic pNET lesions can exhibit pathological heterogeneity, and our study provided further understanding regarding the molecular biological features. We further traced the specification trajectories of malignant cells using both transcriptomic and mitochondrial information, which resulted in the identification of dominant clones with metastatic potential. Subsequently, we established a two-gene signature reflecting the components of dominant metastatic clones following the evolutionary process from primary tumor to metastatic lesion. Our validated the prognostic value of this gene signature in pNET patients and found that it could effectively predict recurrence and survival in patients with pNETs. This is the first prognostic molecular marker for pNETs based on evolution analysis using single-cell data. The sample size of survival analysis in the present study limits the clinical significance of the two-gene gene signature, but this work provided a new idea for further identification of novel biomarkers. Future studies with larger sample sizes are also needed to validate the prognostic value of this two-gene signature.

Because of the low incidence rate and indolent behavior, there are relatively few studies on pNETs. A particular strength of our study was the availability of both primary and metastatic lesions, but there were still some limitations. First, although we acquired high quality single-cell data of more than twenty-thousand cells, they were from a single patient. In terms of the prognostic gene signature presented in this study, we consider that the advantages offered by an enlarged sample size and validation have compensated for this limitation. However, the composition of subpopulations of multiple TME cells, as well as their role in pNETs, still need to be investigated in more cases. Second, G3 neuroendocrine carcinomas are extremely rare and highly aggressive.^56^-^58^ Therefore, findings in this study may not apply to all G3 cases. We also found that the prognostic value of our gene signature was diminished in G3 cases. Third, due to the technical limitation of scRNA-seq, only transcriptome data was obtained in the present study. Approaches such as single-cell genome analysis and single-cell ATAC-sequencing will further advance our understanding of this disease. Overall, these limitations highlight the need for further work to optimize and expand scRNA-seq datasets for pNETs.

In sum, our findings contribute to a better understanding of the spatial and temporal heterogeneity of pNETs through describing the single-cell level gene expression atlas of main cell types in primary and metastatic lesions. Future work further exploring how these findings may be used for prognostic purposes will benefit patients with pNETS.

## Supplementary Material

Supplementary methods and figures.

Supplementary table 1.

Supplementary table 2.

Supplementary table 3.

Supplementary table 4.

Supplementary table 5.

Supplementary table 6.

Supplementary table 7.

Supplementary table 8.
