# Supplementary material for: Single-cell RNA sequencing reveals spatiotemporal heterogeneity and malignant progression in pancreatic neuroendocrine tumor
Source: Int J Biol Sci. 2021 Aug 28;17(14):3760–75. doi: 10.7150/ijbs.61717 (PMC8495381; doi:10.7150/ijbs.61717)
Supplement: Supplementary file 1 — Supplementary methods and figures. [file ijbsv17p3760s1.pdf]

Supplementary Methods

Tissue dissociation and single-cell isolation

scRNA-seq libraries were generated using the 10X Genomics Chromium Controller Instrument and Chromium Single Cell 3' V3 Reagent Kits (10X Genomics, Pleasanton, CA). Briefly, each tissue was cut into cut into 2-3–mm^3 pieces and transported in tubes containing 5 ml RPMI 1640 (Gibco) with 1 mM protease inhibitor (Solarbio) placed on ice. Tissue pieces were incubated for 15 min in a 37 ℃ water bath. Cells were collected through a 40-μm filter into FBS and stored on ice, and red blood cells were removed by RBC lysis buffer (Invitrogen) with 1 unit/ml DNase I. Cells were collected by centrifugation in a 50-ml conical tube at 200 g for 5 min, and were then resuspended in 100 μl of Tyrode's solution. Cell numbers and viability were determined using a Bio-Rad TC20 automated cell counter and 0.4% trypan blue staining.

Single-cell sequencing

Cell concentration was adjusted to 1,000 cells/μl and approximately 10,000 cells per sample were loaded into each channel to generate single-cell Gel Bead-In-Emulsions (GEMs) to barcode the mRNA of 6,000 single cells per sample. After the RT step, GEMs were broken and barcoded cDNA was purified and amplified. The amplified, barcoded cDNA was fragmented, A-tailed, ligated with adaptors, and index PCR-amplified. The final libraries were quantified using the Qubit High Sensitivity DNA assay (Thermo Fisher Scientific) and the size distribution of the libraries was determined using a High Sensitivity DNA chip on a Bioanalyzer 2200 (Agilent). All libraries were sequenced by HiSeq Xten (Illumina, San Diego, CA) on a 150 bp paired-end run. All sequencing data have been uploaded to Gene Expression Omnibus website (GSE162708).

Single-cell RNA statistical analysis

scRNA-seq data analysis was performed by NovelBio Bio-Pharm Technology Co., Ltd. with NovelBrain Cloud Analysis Platform. We applied fastp with default parameter filtering of the adaptor sequence and removed low-quality reads to obtain clean data. Then, the feature barcode matrices were obtained by aligning reads to the human reference genome (GRCh38 ensembl, version 19) using CellRanger v3.1.0. We applied the down sample analysis among samples sequenced according to the mapped barcoded reads per cell of each sample to finally achieve the aggregated matrix. Cells containing fewer than 200 expressed genes or a mitochondria UMI rate above 20% were excluded. Mitochondrial genes were removed from the expression table. The Seurat package (version 2.3.4, https://satijalab.org/seurat/) was used for cell normalization and regression based on the expression table according to the UMI counts of each sample and percent of mitochondrial rate to obtain the scaled data.

Principal component analysis (PCA) and nonlinear dimensional reduction analysis

All the scRNA-seq data from tissues and PMBC were analyzed collectively. PCA and graph-based clustering analysis were performed and visualized by nonlinear

dimensional reduction (t-stochastic neighbor embedding [tSNE], uniform manifold approximation and projection [UMAP]) using the Seurat package (v.2.3.4) for R. Highly variable genes were identified using the 'FindVariableGenes' function with parameters for x.low.cutoff=0.0125, x.high.cutoff=6, and y.cutoff=0.5. PCA was constructed based on the scaled data with the top 2,000 highly variable genes and top 10 principals were used for tSNE construction and UMAP construction.

For subclustering, clusters were selected and PCA was constructed based on the scaled data with the top 2,000 highly variable genes and top 10 principals were used for tSNE and UMAP construction. Utilizing the graph-based cluster method, we acquired unsupervised cell cluster results based on the PCA's top 10 principal components and we calculated the marker genes by the FindAllMarkers function with the Wilcoxon rank-sum test algorithm under the following criteria:1. lnFC > 0.25; 2. pvalue<0.05; 3. min.pct>0.1. In order to identify the cell type, clusters of same cell type were selected for re-tSNE analysis, graph-based clustering, and marker analysis.

CNV estimation and selection of malignant cells

Initial CNVs (CNV_0) were estimated using the inferCNV R package as described in previous studies.[1,2] Then, the extent of the CNV signal, which was defined as the mean of squares of CNV_0 values across the genome, was calculated. Each cell was scored for the extent of the CNV signal and for the correlation between the CNV_0 profile of each cell with the average CNV_0 profile of all cells from the corresponding tumor. Subsequently, putative malignant cells were defined as those with a CNV signal above 0.05 and CNV correlation above 0.5, as previously described.[3]

Pseudo-time analysis

We applied the Single-Cell Trajectories analysis utilizing Monocle2 (http://cole-trapnell-lab.github.io/monocle-release) with DDR-Tree and default parameters. Before Monocle analysis, we selected marker genes from the Seurat clustering results and raw expression counts of the cell-passed filtering. Based on the pseudo-time analysis, branch expression analysis modeling (BEAM Analysis) was applied for branch fate determined gene analysis.

Cell communication analysis

To enable a systematic analysis of cell–cell communication molecules, we applied cell communication analysis based on the CellPhoneDB, a public repository of ligands, receptors, and their interactions (https://github.com/Teichlab/cellphonedb). Membrane, secreted, and peripheral proteins of the clusters of different time points were annotated. The homology relationships between mouse and human genes were downloaded from the Ensembl Biomart database. Significant mean and cell communication significance (P-value <0.05) was calculated based on the interaction and the normalized cell matrix achieved by Seurat normalization.

SCENIC analysis

To assess transcription factor regulation strength, we applied the single-cell regulatory network inference and clustering (pySCENIC, v0.9.5) workflow,[4] using the 20-thousand motifs database for RcisTarget and GRNboost.

Cluster gene enrichment analysis

To characterize the relative activation of a given gene set, such as pathway activation, “Angiogenesis,” and “Fatty Acid Metabolism” as described before, we performed QuSAGE[5] (Quantitative set analysis for gene expression; V. 2.16.1) analysis to calculate the gene enrichment score of each cluster.

Differential gene expression analysis

To identify differentially expressed genes among samples, the function FindMarkers with the Wilcoxon rank-sum test algorithm was used under following criteria: 1. lnFC > 0.25; 2. pvalue<0.05; 3. min.pct>0.1.

Coregulated gene analysis

To discover the gene coregulation network, the find_gene_modules function of monocle3 was used with the default parameters.

GO analysis

Gene ontology (GO) analysis was performed to identify the biological implications of marker genes and differentially expressed genes. We downloaded the GO annotations from NCBI (http://www.ncbi.nlm.nih.gov/), UniProt (http://www.uniprot.org/), and the Gene Ontology (http://www.geneontology.org/). Fisher’s exact test was applied to identify the significant GO categories and FDR was used to correct the P-values.

Pathway analysis

Pathway analysis was used to find the significant pathways of the marker genes and differentially expressed genes according to the KEGG database. We selected the significant pathways using Fisher’s exact test, and the threshold of significance was defined by the P-value and FDR.

Lineage tracing based on mitochondrial mutation

For 10x data, lineage tracing based on mitochondrial mutation was performed following Zhang et al.[6] Briefly, we calculated the alternative allele frequency and the coverage of each position in the mitochondrial chromosome with the help of VarTrix (https://github.com/10XGenomics/vartrix). To build phylogenetic trees based on the mitochondrial mutations, we retained mitochondrial genome position with coverage > 10 and at least 70% cells within the interest cell populations expressed. A cutoff of alternative allele frequency > 0.08 was set to identify subclone mutations in the mitochondrial genome. We calculated phylogenetic trees using RAxML and estimated stable cell lineages.

Patients for survival analysis

In order to validate the prognostic value of gene markers, patient information was retrieved from electronic medical records in two hospitals (Guangdong Provincial People's Hospital, and Sun Yat-sen Memorial Hospital) with the following eligibility criteria: 1) The pathological diagnosis was G1-G2 nonfunctional pNETs; 2) the patients should have received curative surgical treatment; 3) the follow-up time after surgical treatment should be greater than 5 years. Overall, a total of 30 patients underwent curative surgical treatment between January 2006 and January 2016 at Guangdong General Hospital, and a total of 16 patients underwent curative surgical treatment between November 2010 and October 2015 at Sun Yat-sen Memorial Hospital were included in this study. The basic clinical information of the 46 patients were described in the supplementary Table 7. Both the Ethics Committee of Guangdong Provincial People's Hospital and that of Sun Yat-sen Memorial Hospital approved the use of the data for this research with written informed consent being waived as the retrospective nature of this analysis and it involves no risk to subjects.

RNA extraction and quantitative real-time reverse transcription PCR (qRT-PCR)
Total RNA from the fresh frozen tissues was isolated using the Trizol reagent (Takara Bio, Inc., Shiga, Japan) according to the manufacturer's instructions. Total RNA (1 μg) was reverse transcribed using PrimeScript™ RT Master Mix (Takara Bio). Quantitative real-time polymerase chain reaction (qPCR) was performed using the TB Green™ Premix Ex Taq™ II (Tli RNaseH Plus, Takara Bio) and a Roche LightCycler 480 system (Roche, Basel, Switzerland). All primers used for qRT-PCR are listed as followed, and the relative changes were calculated using the $2^{-\triangle\triangle CT}$ method.

Gene Symbol	Forward primer (5' to 3')	Reverse primer (5' to 3')
IGFBP3	TTGCACAAAAGACTGCCAAG	CAACATGTGGTGAGCATTCC
PCSK1	GGACCTCTGAGTATGACCCG	AGCTTTGGCATTTAGCAAGC
CXCL16	GACTCCCCGCCATCGGTTCA	CCCCGAGTAAGCATGTCCAC
SMOC1	AGGTCCTACGAGTCCATGTGT	CACTGCACCTGGGTAAAGG
FGF14	GGAAGGGCAAGCTATGAAAGG	TGGTTCTCGGTACATGGCAAC
GAPDH	GCTAAGCAGTTGGTGGTGCA	TCACCACCATGGAGAAGGC

Immunohistochemistry (IHC)
Immunohistochemistry (IHC) was used to examine the protein level of PCSK1 and SMOC1 in paraffin-embedded primary specimens. Briefly, slides in 4-μm thick were dewaxed and rehydrated with xylene and graded alcohol, respectively. The antigen retrieval procedure was performed using a pressure cooker for 15 min in 10 mM sodium citrate buffer (pH 6.0). Nonspecific binding was blocked by incubation in 3% bovine serum albumin in phosphate-buffered saline. Slides were then incubated with PCSK1 antibody (1:100 dilution, Abcam, CA, USA, number ab191452) or SMOC1 antibody (1:100 dilution, Abcam, CA, USA, number ab200219). Following additional washing with PBS the second antibody was added.
To qualitative the IHC results, the evaluation of IHC staining was reference to the contents of our previous publication.[7] Briefly, IHC score was estimated by using the

following formula: IHC score = staining intensity (0, no staining; 1, light brown; 2, brown; 3, dark brown) × proportion of positively stained cells (0, none; 1, < 25%; 2, 25–50%; 3, 50–75%; and 4, > 75%). All samples were scored by two independent observers in a blinded manner, and a final score ≥6 was considered as a positive expression of the corresponding protein.

Search strategy for retrieving cancer stem cell markers of pNETs

A literature search was performed on July 1st, 2021 in four electronic databases including PubMed, EMBASE, Web of Science, and MEDLINE. The following MeSH terms and their combinations were searched in [Title/Abstract]: [stem and neuroendocrine and (cancer or tumor or tumour or neoplasm or carcinoma or adenocarcinoma)]. In addition, the reference lists of relevant articles were manually searched. The search was limited to English language publications, and there was no restriction on publication year.

Statistical Analysis

Survival curves were compared using the log-rank test, and Kaplan-Meier survival curves were plotted. Survival was calculated from surgical resection to death or last follow-up. Correlation analyses were performed using the spearman correlation. One-way analysis of variance (ANOVA) was used to compare continuous variables of multiple groups, while means between two groups were compared by using t-test or Wilcoxon rank sum test depending on the normality test result. All P values were two sided and $P < 0.05$ was considered to indicate a statistically significant difference.

References

1. Dissecting the multicellular ecosystem of metastatic melanoma by single-cell RNA-seq. *Science (New York, NY)* 2016.
2. Patel AP, Tirosh I, Trombetta JJ, et al. Single-cell RNA-seq highlights intratumoral heterogeneity in primary glioblastoma. *Science* 2014; **344**(6190): 1396-401.
3. Puram SV, Tirosh I, Parikh AS, et al. Single-Cell Transcriptomic Analysis of Primary and Metastatic Tumor Ecosystems in Head and Neck Cancer. *Cell* 2017; **171**(7): 1611-24.e24.
4. Aibar S, González-Blas CB, Moerman T, et al. SCENIC: single-cell regulatory network inference and clustering. *Nat Methods* 2017; **14**(11): 1083-6.
5. Yaari G, Bolen CR, Thakar J, Kleinstein SH. Quantitative set analysis for gene expression: a method to quantify gene set differential expression including gene-gene correlations. *Nucleic acids research* 2013; **41**(18): e170.
6. Zhang Q, He Y, Luo N, et al. Landscape and Dynamics of Single Immune Cells in Hepatocellular Carcinoma. *Cell* 2019; **179**(4): 829-45 e20.
7. Su D, Guo X, Huang L, et al. Tumor-neuroglia interaction promotes pancreatic cancer metastasis. *Theranostics* 2020; **10**(11): 5029-47.

Supplementary Figures

Supplementary Figure S1

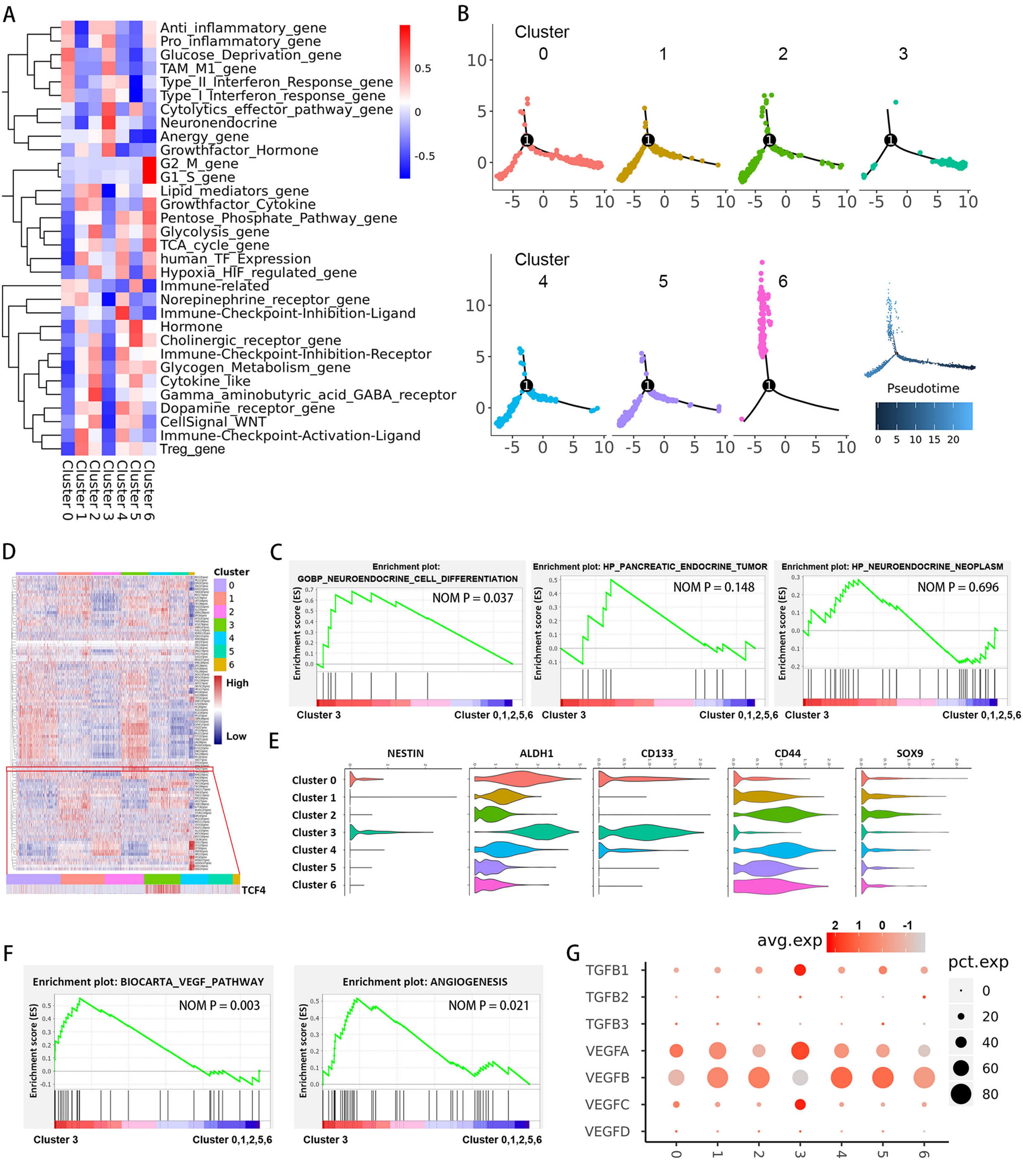

Supplementary Figure S2

A

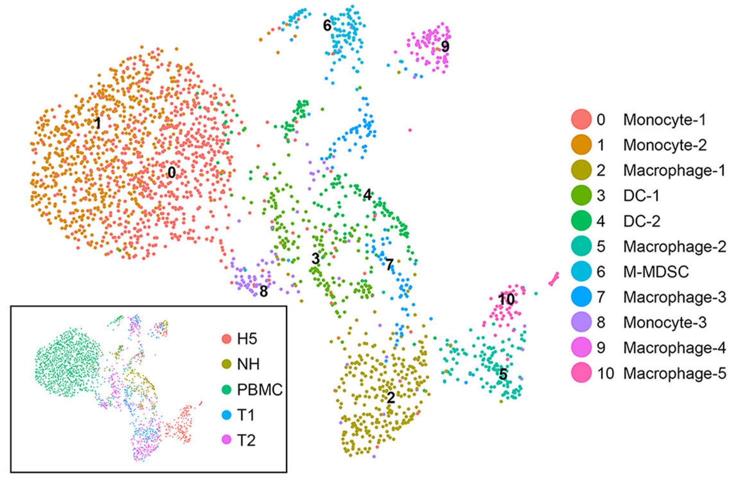

B

Cell type		Samples					Total	Percentage
		NH	PBMC	T1	T2	H5		
0	Monocyte-1	2	631	13	7	0	653	26.45%
1	Monocyte-2	0	598	5	12	0	615	24.91%
2	Macrophage-1	2	0	78	194	6	280	11.34%
3	DC-1	16	0	59	92	2	169	6.84%
4	DC-2	132	9	4	10	3	158	6.40%
5	Macrophage-2	0	0	0	0	128	128	5.18%
6	M-MDSC	5	18	27	65	2	117	4.74%
7	Macrophage-3	35	1	24	35	15	110	4.46%
8	Monocyte-3	3	18	19	41	5	86	3.48%
9	Macrophage-4	24	1	10	26	21	82	3.32%
10	Macrophage-5	0	0	0	6	65	71	2.88%
Total		219	1276	239	488	247	2469	100.00%
Percentage		8.87%	51.68%	9.68%	19.77%	10.00%	100.00%	

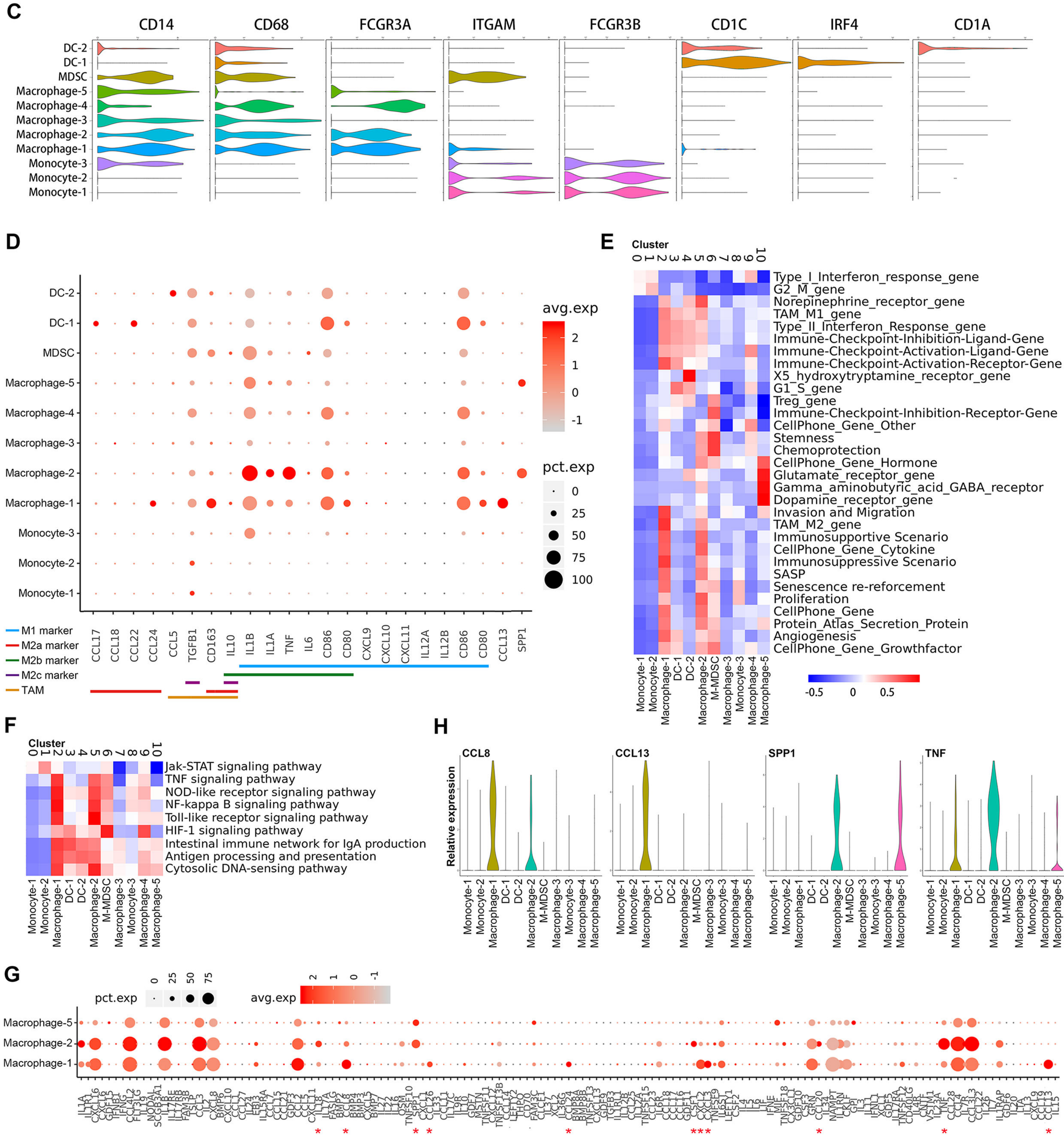

Supplementary Figure S3

A

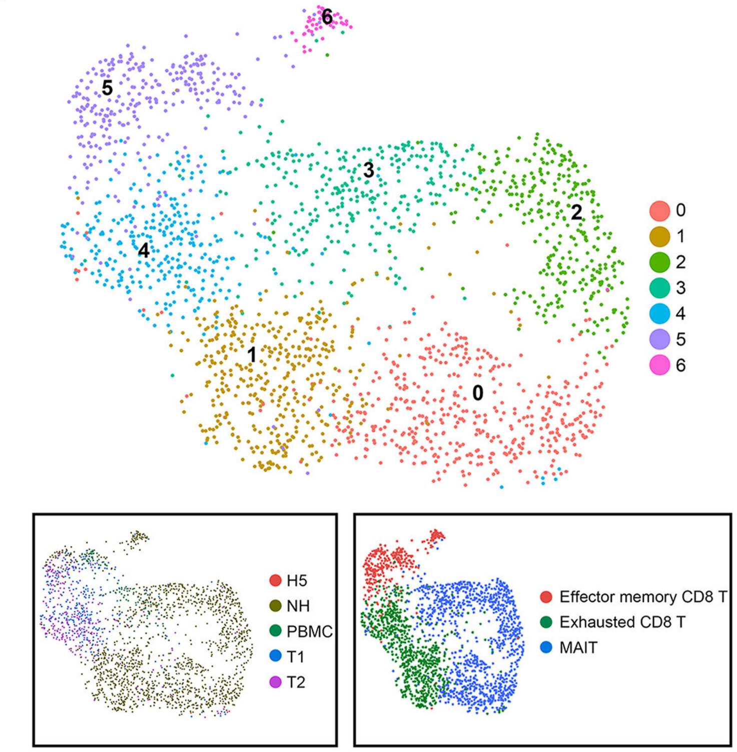

C

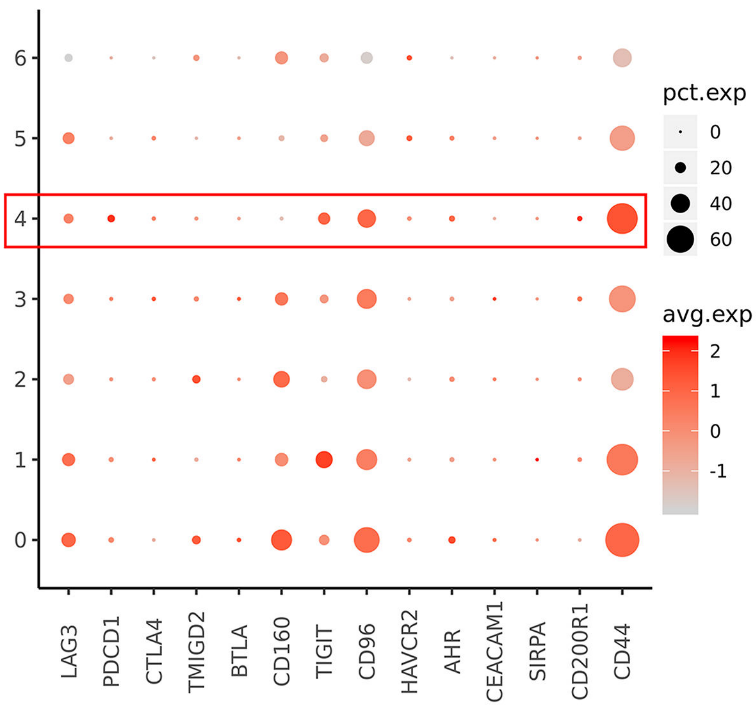

B

Cluster	Samples					Total	Percentage
	NH	PBMC	T1	T2	H5		
0	439	3	14	9	2	467	21.85%
1	391	0	9	11	0	411	19.23%
2	321	0	1	4	1	327	15.30%
3	202	75	18	4	0	299	13.99%
4	11	0	137	149	0	297	13.90%
5	70	48	100	67	3	288	13.48%
6	47	0	1	0	0	48	2.25%
Total	1481	126	280	244	6	2137	100.00%
Percentage	69.30%	5.90%	13.10%	11.42%	0.28%	100.00%	

D

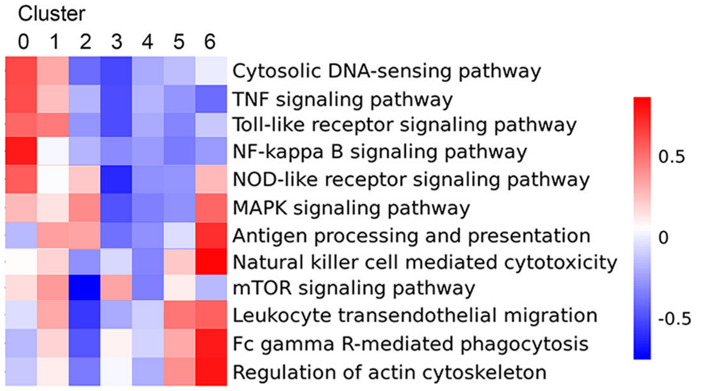

E

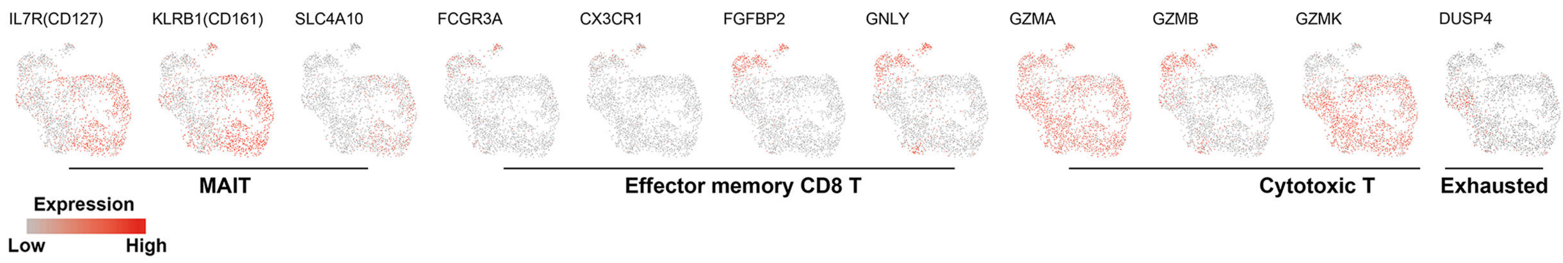

F

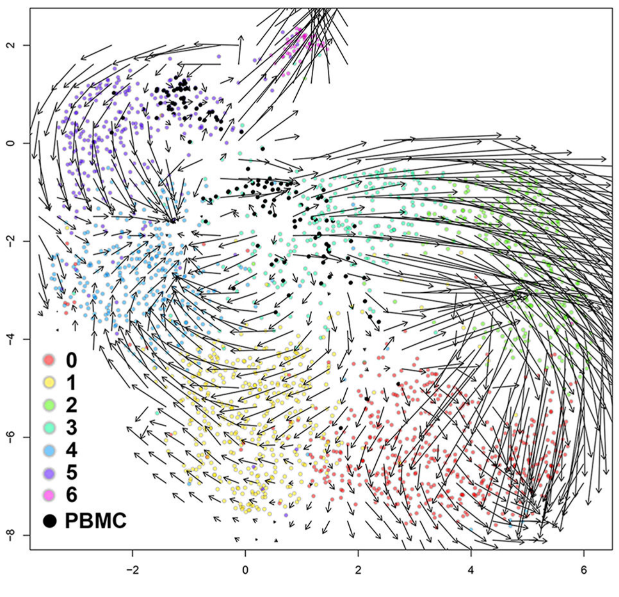

G

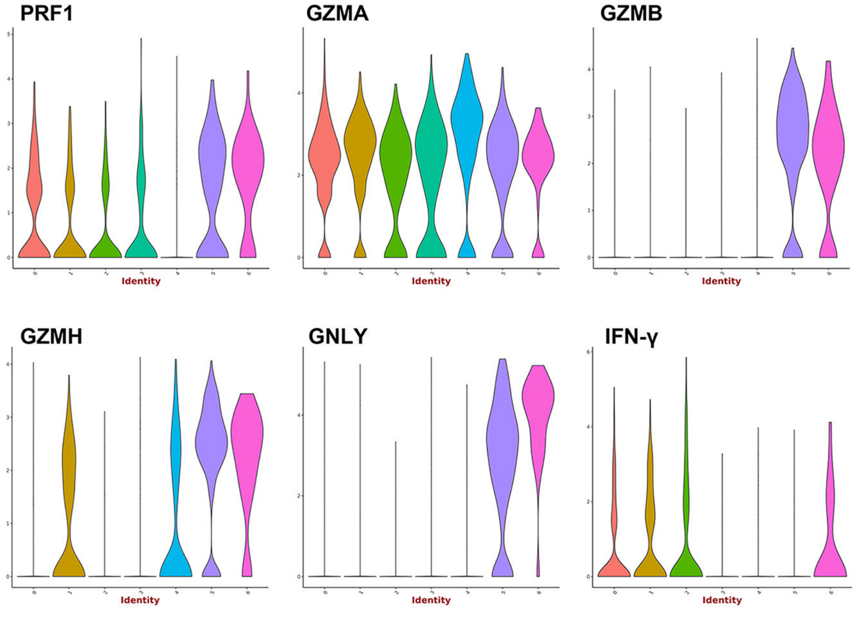

Supplementary Figure S4

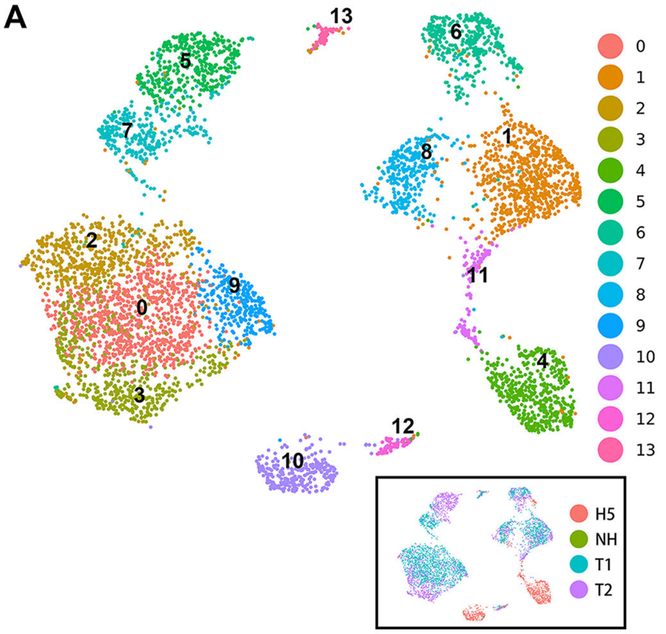

B

Cluster	Samples NH	T1	T2	H5	Total	Percentage
0	0	717	148	1	866	16.59%
1	3	516	258	0	777	14.89%
2	0	449	103	3	555	10.63%
3	0	74	424	1	499	9.56%
4	0	1	2	483	486	9.31%
5	0	38	377	0	415	7.95%
6	2	164	186	32	384	7.36%
7	0	246	92	1	339	6.49%
8	14	184	120	1	319	6.11%
9	1	218	80	2	301	5.77%
10	1	3	6	269	279	5.34%
11	0	15	56	54	125	2.39%
12	0	15	26	27	68	1.30%
13	2	47	16	1	66	1.26%
Total	21	2610	1796	793	5220	100.00%
Percentage	0.40%	50.00%	34.41%	15.19%	100.00%	

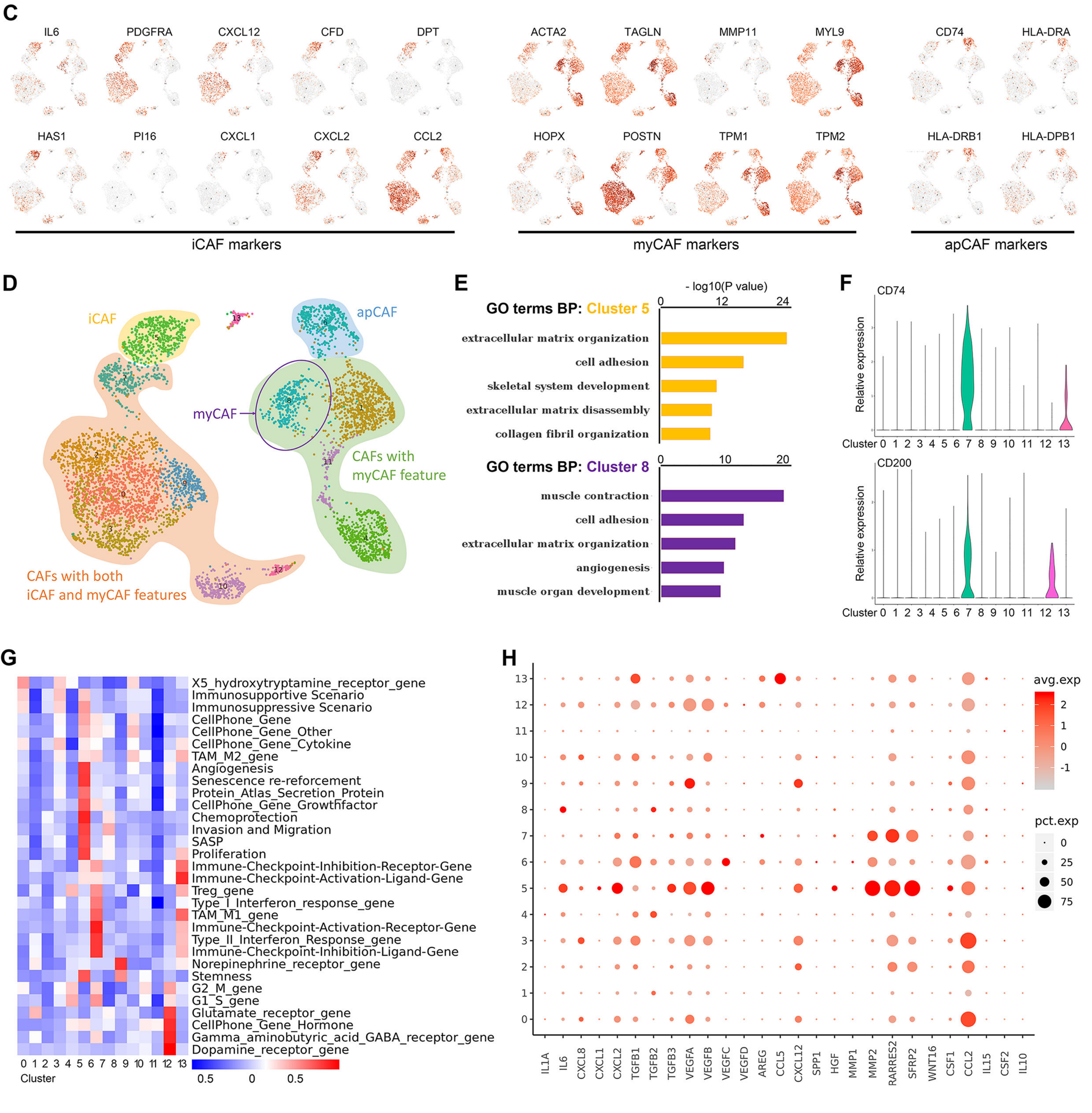

Supplementary Figure S5

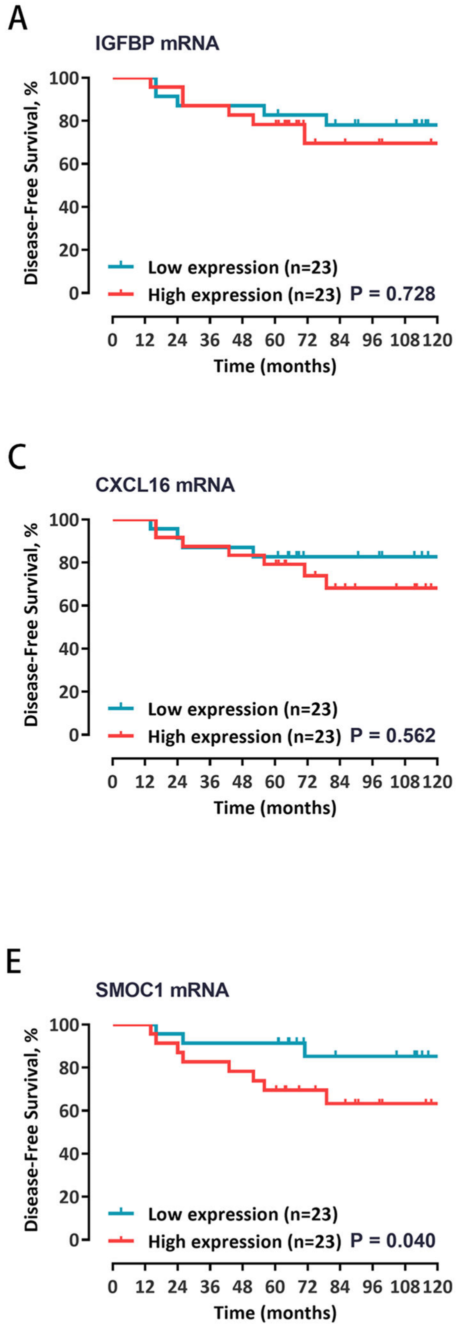

Figure legends of supplementary figures

Figure S1. A. QuSAGE Gene Enrichment Analysis revealed enriched biological processes for specific genes of each malignant subcluster. B. Malignant developmental trajectories of single malignant cells for each subcluster. C. GSEA showing the enrichment of gene signatures in subtype 3 (cluster 3) in comparing with other subtypes. D. Heatmap of SCENIC analysis, showing the relative activity of transcription factors in each malignant subcluster at single-cell level. The blue box highlights the transcription factor TCF4 with high activity specific to subcluster 3. E. Expression levels of NESTIN, ALDH1, CD133, CD44, and SOX9 in each subtype were displayed in violin plots. F. GSEA showing the enrichment of gene signatures associated with angiogenesis in subtype 3 (cluster 3) in comparing with other subtypes. G. Dot plot demonstrates the normalized mean expression of transforming growth factors and vascular endothelial growth factors in each subtype. pct.exp, percentage of cells with expression; avg.exp, average expression level.

Figure S2. A. UMAP plot showing 11 clusters of myeloid cells, colored by cell cluster or sample origin (lower left black box). B. Cell number and percentage of assigned cell types are summarized. C. Dot plot depicting the expression levels of specific markers for monocytes, macrophages, and dendritic cells across clusters. D. Dot plot depicting the expression levels of markers for macrophage subtypes across clusters. E-F. Heatmaps showing the results of QuSAGE analysis. G. Dot plot showing the relative expression of chemokines in macrophage-1, macrophage-2, and macrophage-5. H. Violin plots showing the expression levels of CCL8, CCL13, SPP1, and TNF across clusters. pct.exp, percentage of cells with expression; avg.exp, average expression level.

Figure S3. A. UMAP plot showing 7 clusters of CD8+ T cells, colored by cell cluster, sample origin (lower left black box) or cell type (lower right black box). B. Cell number and percentage of assigned cell types are summarized. C. Dot plot depicting the expression levels of Immune-Checkpoint-Inhibition-Receptor-Genes across clusters. D. Heatmap depicting the enrichment of immune-response related pathways in each cluster based on the result of QuSAGE analysis. E. UMAP plots demonstrating differential expression of selected marker genes of MAIT, effector memory T cell, cytotoxic T cell, and exhausted T cell. F. RNA velocities are visualized on the UMAP projection of in CD8+ T cells using Gaussian smoothing on a regular grid. G. Violin plots showing the expression of PRF1, GZMA, GZMB, GZMH, GNLY, and IFN-γ across clusters. pct.exp, percentage of cells with expression; avg.exp, average expression level.

Figure S4. A. UMAP plot showing 14 clusters of fibroblasts, colored by cell cluster or sample origin (lower left black box). B. Cell number and percentage of assigned cell types are summarized. C. UMAP plots demonstrating differential expression of selected marker genes of iCAF, myCAF, and apCAF. D. UMAP plot showing the functional description of each cluster. E. Enriched biological processes in GO analysis based on marker genes of cluster 5 (upper panel) and cluster 8 (lower panel). F. Violin plots showing the expression of CD74 and CD200 across clusters. G. Heatmap depicting the result of QuSAGE analysis. H. Dot plots depicting the expression levels of SASP-related genes across clusters. pct.exp, percentage of cells with expression; avg.exp, average expression level.

Figure S5. Kaplan-Meier curves of disease-free survival stratified by the median mRNA expression level of IGFBP3 (A), PCSK1 (B), CXCL16 (C), FGF14 (D), and SMOC1 (E).
